# Tetralogy of Fallot: Current surgical perspective

**DOI:** 10.4103/0974-2069.43873

**Published:** 2008

**Authors:** Tom R. Karl

**Affiliations:** Division of Pediatric Cardiac Surgery, UCSF Pediatric Heart Center, San Francisco, USA

**Keywords:** Right ventricular function, residual right ventricular outflow obstruction, residual pulmonary incompetence

## Abstract

Tetralogy of Fallot (TOF) is an important lesion for all pediatric and congenital heart surgeons. In designing the most appropriate operation for children with TOF, the postoperative physiology should be taken into account, both in the short and long term. The balance between pulmonary stenosis (PS) and pulmonary insufficiency (PI) may be critical for preservation of ventricular function. A unified repair strategy that limits both residual PS and PI is presented, along with supporting experimental evidence, a strategy for dealing with coronary anomalies, and comments regarding best timing of operation.

## INTRODUCTION

TOF is an important lesion for pediatric and congenital cardiac surgeons. It was the first cyanotic lesion to be described, and generated some of the initial palliative and definitive cardiac operations. TOF is therefore an excellent model for natural history studies of treated congenital heart disease. Moreover, TOF has had a central role in our understanding of molecular biology, myocardial protection, and inflammation as they relate to congenital heart disease (CHD). Most importantly, although TOF is often lethal if untreated, it now has a good prognosis with timely surgical intervention, even if a uniformly excellent result remains elusive.

The ideal TOF repair should be suitable for children of all ages, and should provide good relief of right ventricular outflow tract obstruction (RVOTO) to prevent progression of right ventricular hypertrophy. There should be complete atrial and ventricular septation, with avoidance of ventriculotomy and circulatory arrest. Preservation of pulmonary valve (PV) and tricuspid valve (TV) function and biventricular contractility would also be considered fundamental, along with minimal early mortality and morbidity. In the current era, in which the immediate outcome of TOF repair is good employing diverse surgical strategies, the goal of treatment should include the avoidance of long-term complications and a low probability of early and late reoperations. A good neurodevelopmental and functional status and quality of life could complete the expectations.

Lillehei's original TOF repairs (whether with cross circulation or cardiopulmonary bypass(CPB)) generally employed a large right ventricular incision for access to both the RVOTO and ventricular septal defect (VSD). The relatively favorable long term results have been noted in numerous studies.[1–3] However, it has become evident that myocardial injury, coronary injury, right ventricular dysfunction, and arrhythmias are all related in part to the right ventricular incision, especially in the context of transannular patching and free pulmonary insufficiency (PI). In some patients therefore the late outcome will be compromised. Today we have a better understanding of some of the events leading to late right ventricular failure, and perhaps the technical ability to improve the results for the next generation of children undergoing repair, which constitutes the subject of this paper.

The traditional thinking regarding post repair TOF physiology has emphasized two points. The first is that postoperative RVOTO is undesirable, to the point that many teams would consider revision of a repair in the setting of a right ventricle (RV) / left ventricle (LV) pressure ratio > .75. The second traditional belief is that postoperative PI is well tolerated and that only very significant RV dilation is problematic. Both concepts have been challenged in recent years. PI is exacerbated by loss of distal compliance and lessened or prevented by proximal resistance. Free PI causes right ventricular dilation that in turn compresses the LV. The degree of PI depends in part on pulmonary arterial compliance, and the location of resistance relative to valveless RV-PA junction.[4]

The functional reserve and myocardial contractility of the right (and possibly left) ventricle decrease with chronic PI, detectable in experimental models as early as 3 months after the onset of PI.[5] Such physiology is well tolerated in the majority of patients for a prolonged period of time, but some will eventually experience decreased exercise tolerance and progressive RV dilation and failure.[6,7] This in turn predisposes the patient to late life-threatening ventricular arrhythmias and sudden death.[6,8] Recent studies have focused on the evaluation of the effects of PI on ventricular function and propose less restrictive indications for PV replacement.[9–14] Not all patients will benefit, and the timing and indications are controversial. Assessing the severity may require sophisticated tools, Tissue Doppler imaging of tricuspid annular motion during dobutamine stress echocardiography, for example, is useful for estimating contractility reserve, which may be relevant for timing of PVR.[15] Diastolic function may be as important as systolic function in timing.[16] There is no consensus on the best tools for assessment, nor their relative importance in decision making. Magnetic resonance imaging (MRI), brain natriuretic peptide (BNP) levels, QRS duration, presence of arrhythmia, anatomy of the RV, and other factors have all been proposed.[17–22] Despite our efforts, not all patients will improve following revisional surgery.

Following TOF repair, there is almost always residual RVOTO, seen immediately or over time. Furthermore, the degree of RVOTO may be inversely proportional to the grade of PI. With an isolated pressure load, increases in cellular hypertrophy, microtuble density, and interstitial collagen formation will lead to decreased contractility over time. In balancing PS and PI, it would be useful to know which is worse for the RV and LV over the long term: isolated PI or combined PSPI? Should we try to limit PI by accepting more PS at the initial repair? Can we eliminate both problems using appropriate techniques?

## EXPERIMENTAL METHOD

**Figure 1:**
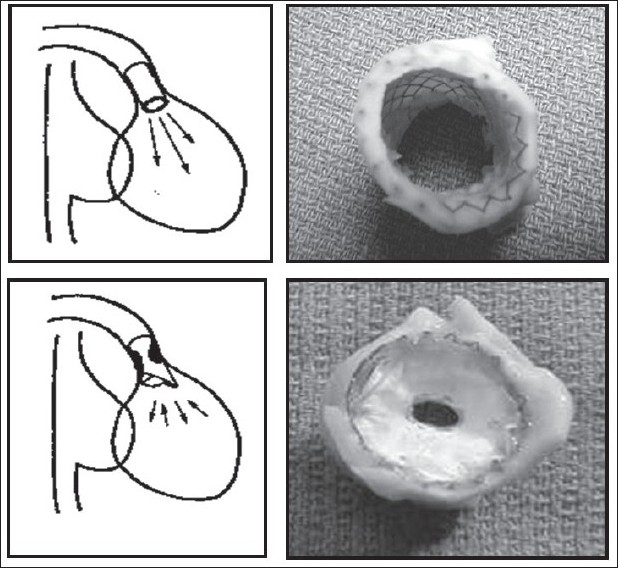
Creation of a chronic porcine model of PI (upper frames) and combined PS/PI (lower frames), achieved with percutaneous delivery of transannular stents (with and without a PTFE diaphragm) into the RVOTO (from Kuehne et al, 2005, used by permission)

**Figure 2:**
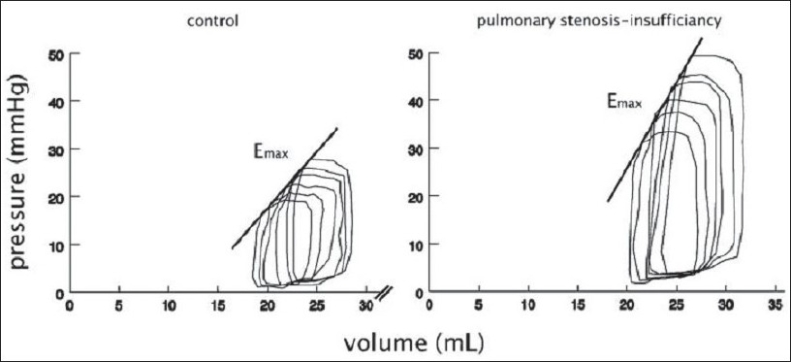
Representative right ventricular pressure-volume loop of a pig with combined pulmonary stenosis and insufficiency and a control animal (measured at rest) at 3 month follow-up. Emax, slope of end-diastolic pressure-volume relation. (from Kuehne et al, 2005, used by permission)

The UCSF interventional cardiology team have created a non surgical porcine model to assess the effects of chronic combined PSPI on RV and LV function over time, looking at both anatomic and functional changes[23] [Figure 1]. Growing pigs had customized transcutaneous stents placed to induce combined PSPI. Assessment was done within 2 days of intervention and 3 months later, looking at indices of systolic function (stroke volume, ejection fraction, and cardiac functional reserve), myocardial contractility (slope of the end-systolic pressure-volume and change in pressure over time-end-diastolic volume relationship), and diastolic compliance. MRI was used to quantify PI and ventricular volumes. Conductance catheters were used to obtain indices of the cardiac functional reserve, diastolic compliance, and myocardial contractility from pressure-volume relations acquired at rest and under dobutamine infusion [Figure 2]. The data were compared with data from controls and animals with isolated PI.[24]

After 3 months of PSPI, indices of RV systolic pump function were decreased at rest and failed to increase appropriately during dobutamine stress. Impaired systolic pump function was associated with decreased RV diastolic compliance but enhanced RV myocardial contractility. These findings contrasted markedly with the effects of isolated PI observed in a similar model.[25] In addition, PSPI resulted in a smaller regurgitant fraction, more RV hypertrophy, and a smaller increase in RV volumes than did isolated PI.

Stenosis, in addition to insufficiency, seemed to have been beneficial by promoting hypertrophy, limiting RV dilatation, and enhancing myocardial contractility. These beneficial effects of short- to intermediate-term stenosis on RV myocardial contractility raise many questions in light of the known adverse effects of long-term severe PS on RV function. Our physiological findings need to be confirmed in long-term studies and for different degrees of PSPI. However, this also brings into question the long-held belief that stenosis, even mild, is detrimental to ventricular function while insufficiency is well tolerated.

## RELEVANCE FOR SURGICAL TECHNIQUE FOR TOF

**Figure 3:**
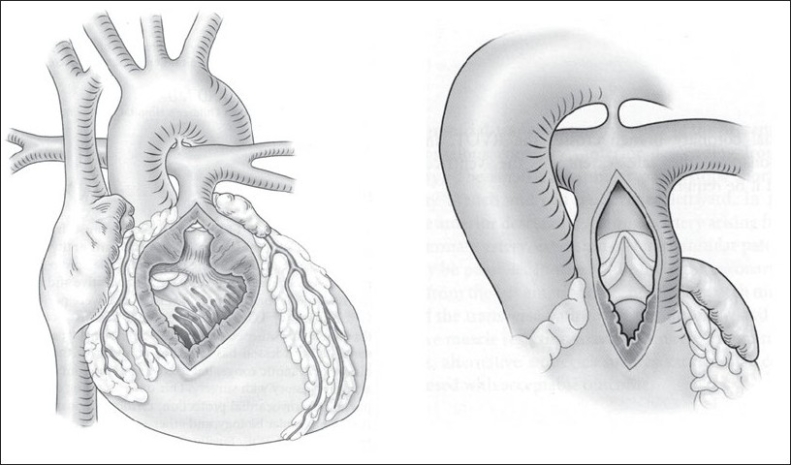
Comparison of the classical transventricular approach (left) and the transatrial-transpulmonary approach (right). In the latter strategy the RV incision is limited to what is required to relieve the RVOTO, with VSD closure and RVOT resection performed via the TV and PA

The acute change from a pressure-loaded to a volume-loaded RV, in addition to the right ventriculotomy, can adversely affect the performance of the RV in the immediate postoperative period.[25,26] Our understanding of postoperative TOF physiology leads us directly to the TA-TP tetralogy repair as a possible solution. The TA-TP approach involves RV outflow tract (RVOT) resection via the TV and PV, and VSD closure via TV.[27] We try to complete the repair without division of the annulus, although if this is not possible we perform a limited ventriculotomy, which is reconstructed with a transannular patch. We can usually avoid a large ventriculotomy into the RV body, limiting it to the outlet portion of RV or infundibulum, which has a relatively minor functional contribution to RV stroke volume[28] [Figure 3]. The TA-TP approach has resulted in significantly less RV dilatation and better preservation of contractility at 10 years follow up.[29,30] Since the description of the pulmonary cusp augmentation technique by Sung and associates[31] in 2003, we have adopted this technique for most patients requiring a transannular patch.

**Figure 4:**
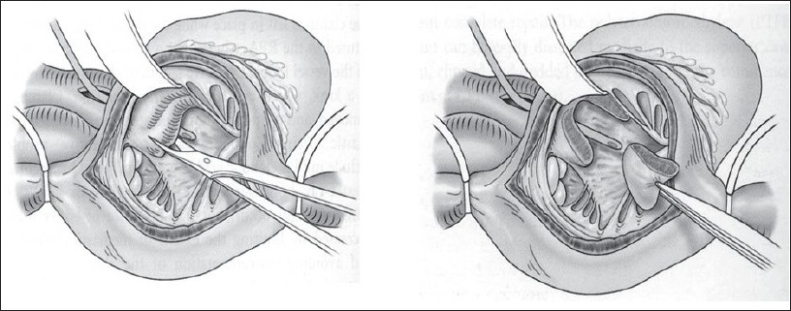
Resection of the parietal extension of the infundibular septum, using a trans tricuspid approach. A Hegar dilator has been passed from the PA into the RV to demonstrate the outlet

Our current operative technique is outlined in [Figures 3 and 4]. A patch of autologous pericardium is harvested and treated with 0.2% glutaraldehyde for 10 minutes. CPB is established (32-34°C) through bicaval and aortic cannulation. Systemic to PA shunts, if present, are ligated after the beginning of CPB. The heart is arrested with antegrade cold blood cardioplegia. The right atrium is opened longitudinally, and the left heart is vented through the foramen ovale or atrial septal defect. A longitudinal incision is made in the main PA and extended to the annulus of the PV. Commissural fusion or tethering of the leaflets to the PA wall is addressed with simple incision. Excision of the parietal extension of the infundibular septum is performed through the TV and PA as required [Figure 4]. Hegar dilators are passed through the TV toward the main PA. If the annular Z score is < –2 (referenced to predicted norm for the patient's weight) the annulus is divided.[32]

In the majority of cases the pulmonary valve will be bicuspid with location of the commissures at the 3 and 9 o'clock positions. An incision is made in the middle of the anterior cusp and then extended to the right ventricular free wall for approximately 10 to 15 mm. When the commissures are located at the 6 and 12 o'clock positions or the commissures are anterior and posterior but located off the midline, we divide the pulmonary valve at or near the anterior commissure to preserve as much valve tissue as possible, as described by Sung *et al*.[31] The division of the remaining obstructing muscular and fibrous bands is performed through the ventriculotomy. The VSD is closed through the TV. The foramen ovale is closed, the heart is de-aired, and the clamp is removed.

**Figure 5:**
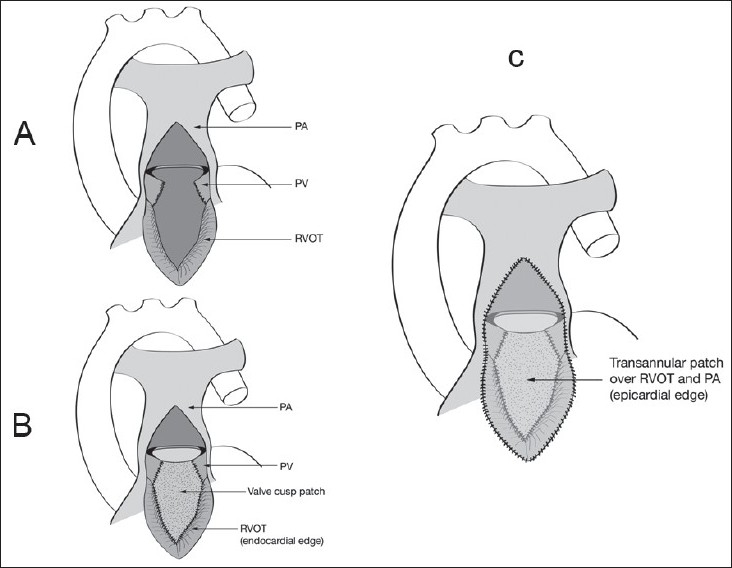
(A) Pulmonary cusp augmentation repair technique. A transannular incision has been created according to calibration of the RVOT diameter (see text). The incision divides the anterior pulmonary valve leaflet, although the exact location will vary with the valve orientation and morphology. (B) Pulmonary cusp augmentation repair technique. A triangular patch of glutaraldehyde-preserved autologous pericardium is sutured to the epicardial edge of the RVOT incision and to the divided edges of the valve leaflet. The leaflet dimensions are based on the caliber of a normal pulmonary valve diameter and should provide sufficient free edge diameter to ensure coaptation with the native valve remnant. (C) Pulmonary cusp augmentation repair technique. A second larger oval patch of the same material is sutured to the epicardial aspect of the RVOT incision and to the edges of the pulmonary arteriotomy, creating a sinus anterior to the reconstructed leaflet

For transannular reconstructions (i.e. cases in which the PV cannot be spared) a triangular glutaraldehyde-treated autologous pericardial patch is sutured to the endocardium from the most inferior aspect of the right ventriculotomy all the way up to the hinge point of the anterior cusp and then along the divided edge of the valve on either side. When the anterior cusp is small or the anterior commissure is located at the 12 o'clock position, the patch is anchored to the main PA. In patients in whom the commissure is eccentric, the patch is sutured to the main PA on one side and to the free edge of the leaflet on the other side. Then a second patch is used to close the RVOT and the pulmonary arteriotomy, thus recreating a sinus over the augmented valve leaflet [Figure 5].

In our initial study of this technique, beginning in November 2001, 43 patients with TOF and 2 patients with isolated pulmonary valve stenosis had relief of RVOTO with either a transannular patch plus pulmonary valve cusp augmentation (n = 18) or a transannular patch alone (n = 25).[26] The median age (5.3 vs 3.2 months; P = .09) and weight (6.4 vs 5.2 kg; P = .3) were similar for the cusp augmentation and transannular patch groups, respectively. The diameter of the pulmonary valve annulus (6.4 vs 6.0 mm; P = .57) and the McGoon index (1.47 vs 1.69, P = .75) were also similar. The mean aortic clamp time (48 ± 18 minutes vs 52 ± 19 minutes; P = .46) and median cardiopulmonary bypass time (89 vs 91 minutes; P = .9) did not differ. Patients with a pulmonary valve cusp augmentation had a shorter duration of intubation (P < .001) and intensive care unit stay (P < .001). Thirteen patients with a transannular patch and 1 patient with a pulmonary valve cusp augmentation required inotropic support for more than 72 hours (P = .001). Discharge echocardiograms demonstrated moderate or severe pulmonary insufficiency in 5 patients with a pulmonary valve cusp augmentation and in 21 patients with a transannular patch (P < .001). At 7.5 months, 3 patients (17%) with a pulmonary valve cusp augmentation had progression of pulmonary insufficiency.

Of the 18 patients who underwent pulmonary valve cusp augmentation, 1 was lost to follow-up. Two patients required reintervention. One patient presented to the hospital 3 months postoperatively with a delayed pericardial effusion and required a pericardial window. The second patient was symptomatic with persistent subvalvar pulmonary stenosis. She underwent operative RVOT muscle bundle resection 2 years after her original repair. This patient had moderate PI at discharge, and the degree of PI did not change during follow-up. At a median follow-up of 7.5 months (range 0-29 months), 6 of the 17 patients (35.3%) had moderate or severe PI at follow-up. Three patients (17%) who underwent pulmonary valve cusp augmentation had progression of PI when compared with the degree of PI at discharge. Similar improved outcomes have been demonstrated in a larger cohort by Sung *et al*, employing this technique.[31]

## DISCUSSION

Several groups have described strategies to limit the size of the right ventriculotomy and reduce the incidence of PI. Valved homografts and xenografts were proposed as the most anatomic and physiologic way to resolve RVOT obstruction, but all will eventually need replacement because of patient growth and conduit deterioration.[33] The use of a monocusp valve created from pericardium, xenograft valve cusps, fascia lata, autologous pulmonary artery wall, or PTFE has been shown to decrease short-term PI.[34–36] However, the monocusp has limited durability.[33,34] Many groups have abandoned the monocusp because they did not think that it provided a significant advantage for the long term. Seventeen percent of our patients had progression of PI on short-term follow-up (median 7.5 months). In Sung and colleagues series, 2 of the 18 patients also had progression of PI at the 10-month follow-up.[31] We will continue to follow these patients to determine whether this reconstruction is durable or not.

The current technique may be different, in that it preserves the native hinge mechanism of the valve cusps, especially when the pulmonary valve is bicuspid and the commissures are located in the 3 and 9 o'clock positions. The use of native valve tissue also offers the theoretic potential of growth. The pulmonary kneecaps technique can be used in the majority of patients with TOF and pulmonary stenosis who require division of the pulmonary valve annulus for relief of RVOT obstruction. In our experience it cannot be applied only in a certain subgroup of patients who have a small pulmonary annulus with hypoplastic inadequate native pulmonary valve tissue. In this group of patients the TA-TP repair with a minimal right ventriculotomy and closure of the RVOT with a simple transannular patch is preferred.

Despite the limitations of our study, the augmentation of the pulmonary valve cusp with autologous pericardium is simple, does not significantly prolong the operation, reduces the degree of PI in the immediate postoperative period, and improves the early outcome after TA-TP TOF repair requiring a transannular patch. When technically feasible, it has become our preferred technique for RVOT reconstruction in patients with TOF and in patients with pulmonary stenosis who require surgical intervention.

## TOF WITH ANOMALOUS CORONARY ARTERIES

**Figure 6:**
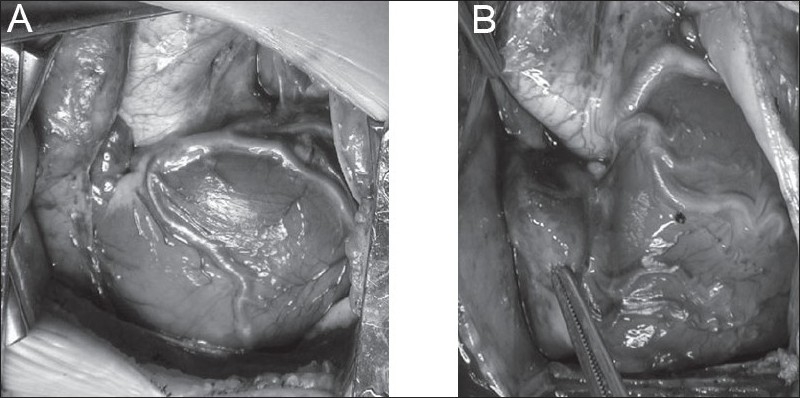
(A) LAD from the RCA, (B) all coronaries arising from the right sinus. Both situations would be problematic for a transventricular repair, but both patients had successful transatrial-transpulmonary repairs

**Figure 7:**
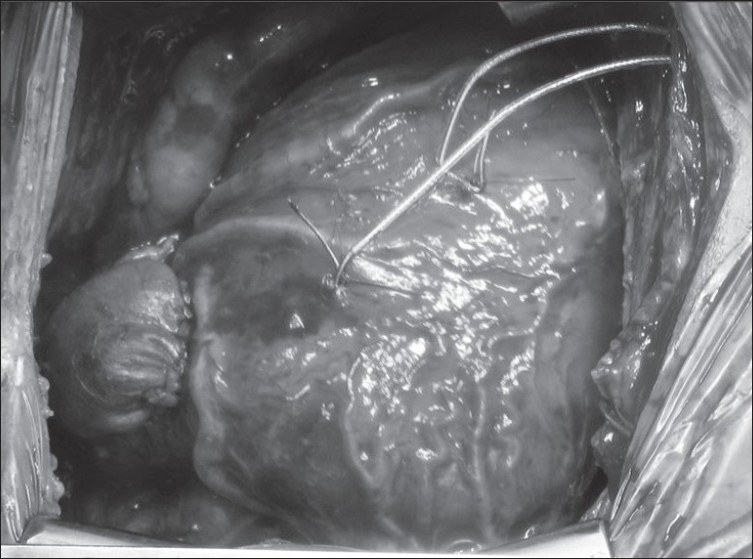
TOF with RCA arising from LAD and crossing the RVOT. A transatrial-transpulmonary repair has been performes with a transannular patch stopping short of the anomalous coronary

Coronary anomalies occur in between 5% and 12% of patients with TOF.[13–15] The most commonly encountered patterns are left anterior descending (LAD) from right coronary artery (RCA), RCA from left coronary artery (LCA), RCA from LAD, large conal artery from RCA, and single RCA [Figure 6]. In the current era the coronary anatomy can be effectively imaged with standard 2D echocardiography. These abnormalities of epicardial distribution are generally not associated with myocardial ischemia. Their main importance is related to surgical repair, at which time coronary injury could be inflicted during the ventriculotomy or the RVOT resection. One technical advantage of the TA-TP approach is the possibility of undertaking repair even when a major coronary artery branch crosses the infundibulum, precluding a conventional transventricular repair[32] [Figures 5 and 6]. We have employed the TA-TP repair in such cases with and without a concurrent PV leaflet repair as described above, and the results (compared to cases with normal coronary patterns) were presented nearly a decade ago, with a continued favorable experience to date [Figure 7]. In our initial experience at the Royal Children's Hospital, Melbourne, the TA-TP approach was used in 611 consecutive repairs, 36 (5.9%) of which were associated with a surgically relevant coronary artery anomaly. The median age and weight of the patients at repair were 23 months (2.8-170 months) and 9.9 kg (5.2-41 kg), respectively. Anomalies included LAD from RCA or single RCA (n = 22), RCA from LCA or LAD (n = 8), and large RCA conal branch (n = 6). The TA-TP approach was successful in 34/36 cases, in 25 of which placement of a limited transannular patch was necessary. Two patients had a RV-PA conduit as a result of proximity of the coronary branch to the pulmonary anulus and our inability to adequately relieve the RVOTO. There were no early or late deaths in the anomalous coronary patients. Mean right ventricle-pulmonary artery gradient at last follow-up was 19 mm Hg (95% confidence interval 14.5-24 mm Hg), compared with 15 mm Hg (95% confidence interval 12.5-17.5 mm Hg) for patients with normal coronary arteries (p = .3). Actuarial freedom from reoperation at 120 months was 96.5% (95% confidence interval 79.8%-99.5%) and was similar for between patients with and without coronary artery abnormalities (p = .92). Thus this series of patients with anomalous coronary arteries demonstrates the same short and long term results after repair as seen in the remainder of our cohort with TOF.

## TIMING OF SURGERY

The best age for TOF repair remains controversial, and the strategy employed may influence the technique of operation. TOF can be repaired at any age with a low operative risk in units equipped to deal safely with complex heart surgery in infants. The TA-TP strategy particularly is easier and more reliable in infants 2 - 6 months of age than in neonates, but is certainly possible at any age. In our unit since November of 2001, we have used the TA-TP approach for TOF repair, following the policy set forth by Mee *et al* at the Royal Children's Hospital, Melbourne.[27] Neonates with an unacceptable level of hemoglobin saturation and/or hypercyanotic spells may be palliated with a modified Blalock-Taussig shunt. Although there is documented morbidity and interval mortality with this approach there are centers that report excellent outcome and a risk of palliation that approaches 0%.[27,35,36] Proponents of primary neonatal repair cite factors such as prevention of time related end organ damage from cyanosis, removal of stimulus for RV hypertrophy and fibrosis, improved lung development (vascular and alveolar), avoidance of deleterious effects and risks of palliative shunts, and psychosocial-economic issues (for the family and care givers). Most of these arguments have some merit, although the difference between neonatal and later infant repair seems difficult to measure. Many surgeons employing a neonatal tetralogy repair strategy still employ a transventricular approach, with a transannular valveless RVOT patch reconstruction, despite the now well documented effects of these stratagies on the RV. Furthermore, an interatrial communication is usually maintained post operatively, creating a situation in which neither PO_2_ nor cardiac output can be guaranteed. Although survival statistics are generally favorable, most published neonatal TOF series have not had a long enough followup time to fully assess the effects of free PI.[37–39]

The argument is often put forth as primary neonatal repair versus palliative systemic to pulmonary arterial shunt with later repair. However, virtually every published report of infant or neonatal tetralogy repairs excludes or mentions patients who had a palliative shunt rather than a primary repair. Therefore even the strongest proponents of neonatal primary repair still consider the modified Blalock Taussig shunt (MBTS) a requirement for selected cases, such as anomalous coronaries, multiple VSDs, generalized critical illness, rehabilitation of small or distorted PAs, prematurity and low birth weight, need to establish a volume load to prevent diastolic dysfunction after repair (older children), and even religious objection to blood transfusion. In fact, most tetralogy patients (probably 75%) do not require any surgical treatment in the neonatal period. The important argument therefore is not “primary repair vs shunt” but “neonatal vs non-neonatal” elective primary repair”.

**Figure 8:**
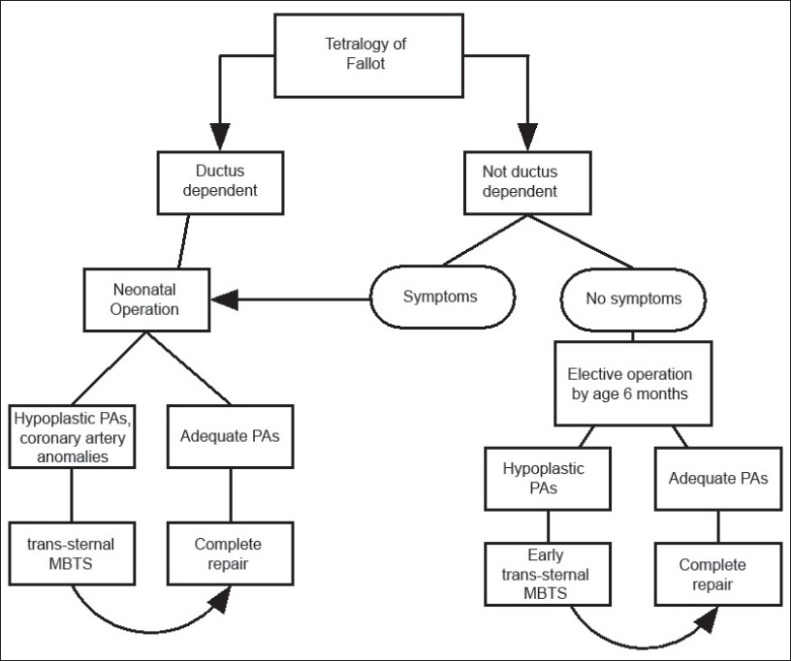
Current protocol for timing of repair for neonates and infants with TOF. In practice, most patients will undergo elective repair by 3-4 months of age, or sooner if indicated clinically. The shunt strategy is reserved for selected cases with unfavorable anatomic features or compromised clinical condition (see text)

Newborns with CHD are known to be susceptible to abnormal brain development and widespread CNS abnormalities, which may be exacerbated following neonatal operations utilizing CPB, especially with deep hypothermia-circulatory arrest (DHCA). Arguments for neonatal repair in the current era should include a detailed assessment of neurodevelopmental status, which rarely appears in outcome data for “early” tetralogy repair, even if “prevention of cerebral injury” is widely cited as a justification for early repairs. Newborns are known to be more susceptible to brain injury such as periventricular leukomalacia in the perioperative period, especially with the use of DHCA.[40,41] Nonetheless, the majority of neonatal tetralogy repairs (even in the current era) have employed a CPB strategy involving DHCA and-or low CPB. It is clear that the frequency and severity of brain injury (pre and postoperatively) for all neonatal heart surgery depends on the sensitivity of tools used to assess it.[42] It is also evident that for cyanotic lesions, cerebral oxygen delivery may actually be (temporarily) worse immediately after repair than before repair, at the most vulnerable time for cerebral injury in the child's lifetime. This is generally not the case with non-neonatal repair. Therefore if neonatal tetralogy surgery can be safely delayed, and if other outcome variables would look similar with an operation at 3-6 months of age (with or without a MBTS), then we would want to follow a strategy of elective non-neonatal primary repair. The solution will vary from unit to unit, and our thinking remains in evolution on this point. Our current preference for timing of operation is presented in [Figure 8]. Non-neonatal primary elective repair (3-6 months) is preferable to a staged procedure involving palliative shunts in most patients. Cyanotic neonates who are suitable anatomic and physiologic candidates will also have a primary repair. There are many excellent cardiac units (all with favorable neonatal experience in other lesions) in which elective primary repair in newborns with TOF is still avoided (Toronto, Melbourne, Chicago, Houston, New Delhi, San Francisco, Buenos Aires, Seoul, London, Auckland, Tokyo, Santiago, Madrid, Paris, etc.)

## CONCLUSION

Considerable progress has been made in the repair strategy for TOF, based on our improved understanding of post repair physiology. There are important implications for timing as well as technique of surgery, and continued evolution is expected.
